# Associations between comprehensive dietary composition and kidney stone risk: insights from a nationally representative survey

**DOI:** 10.3389/fnut.2025.1624543

**Published:** 2025-10-31

**Authors:** Bo Li, Feng Li, Xi Xie, Chen Hui Xiang, Meilin Li

**Affiliations:** ^1^Department of Urology, Clinical Medical College and The First Affiliated Hospital of Chengdu Medical College, Chengdu Medical College, Chengdu, China; ^2^Department of Gynecology, Clinical Medical College and The First Affiliated Hospital of Chengdu Medical College, Chengdu Medical College, Chengdu, China

**Keywords:** dietary composition, kidney stone, National Health and Nutrition Examination Survey, dietary patterns, restricted cubic spline

## Abstract

**Background:**

This study aimed to identify key dietary components exhibiting significant associations with risk of kidney stone (KS).

**Methods:**

This is a cross-sectional analysis that included data from the National Health and Nutrition Examination Survey (2007–2020) based on 26,372 qualified individuals, who provided self-reported information regarding KS and dietary composition over two days. The relationship between the risk of KS and dietary composition were evaluated using weighted multivariate logistic regression models and restricted cubic spline (RCS) models.

**Results:**

Through weighted multivariate logistic regression model, daily consumption of citrus, melons, and berries (OR = 0.81, 95% CI = 0.70–0.93), tomatoes (OR = 0.78, 95% CI = 0.61–0.99), milk (OR = 0.84, 95% CI = 0.78–0.91), total dairy (OR = 0.89, 95% CI = 0.84–0.94), and alcoholic drinks (OR = 0.88, 95% CI = 0.84–0.92) were associated with a lower likelihood of KS development, while daily consumption of added sugars correlated with an elevated probability of KS occurrence (OR = 1.01, 95% CI = 1.00–1.01). Restricted cubic spline analysis found that total fruits, total vegetables, total protein foods, total grains, total dairy, oils, solid fats and added sugars and the risk of KS were in a curvilinear relationship adjusted for age, sex, race, marital status, BMI (body mass index), physical activity in recreational time, smoking status, hypertension, diabetes mellitus (DM). (p overall and p non-linear <0.05).

**Conclusion:**

This cross-sectional study elucidates intricate curvilinear associations between dietary components and the risk of KS. The consumption of citrus, melons, berries, tomatoes and milk was associated with a significantly lower risk of KS. Our findings highlight the need for prospective studies to confirm these potential protective relationships.

## 1 Introduction

Renal calculi represent a prevalent urological affliction, exhibiting a global surge in incidence (1). In the United States, the prevalence of kidney stone (KS) approximates 10.6% among males and 7.1% among females, necessitating clinical intervention for the majority of affected individuals (2). Studies have indicated that the 5-year recurrence rate of renal calculi surpasses 50% (3). Beyond renal colic, urinary tract obstruction, and infection, the elevated incidence and recurrence of renal calculi substantially escalate long-term healthcare expenditures, thereby placing a considerable strain on socioeconomic resources (4). Studies have shown that underscores the pivotal role of dietary consumption in renal calculi formation (5, 6). Consequently, investigating dietary-based preventive strategies holds significant clinical and public health implications.

The influence of dietary components on renal calculi risk frequently manifests as a complex, paradoxical effect. The potassium and citric acid abundant in fruits can mitigate the risk of lithiasis by alkalinizing urine and impeding the crystallization of calcium oxalate (7, 8). The dietary fiber present in vegetables and fruits has the potential to diminish the risk of renal calculi (9, 10). Conversely, certain vegetables (11) with elevated oxalate concentrations or excessive consumption of vitamin C-rich fruits (12) may augment urinary oxalate excretion, thus fostering lithogenesis. Similarly, variations in dietary protein origins markedly influence the propensity for lithiasis. Consumption of plant-derived proteins correlates with reduced urinary uric acid excretion and diminished acidification (13), whereas elevated animal protein (13, 14) intake may synergistically heighten lithiasis risk by augmenting urinary uric acid, calcium, and oxalate excretion. However, prior research has predominantly focused on isolated nutrient analyses, and due to limited sample sizes and inadequate control of confounding variables, the findings have exhibited substantial heterogeneity. Furthermore, conventional dietary assessment methodologies struggle to precisely quantify compounds within intricate food combinations, thus impeding comprehensive analyses of the correlation between dietary and renal calculi.

To mitigate the above limitations, this study integrated nationally representative data from the National Health and Nutrition Examination Survey (NHANES) with the standardized conversion framework of the Food Pattern Equivalency Database (FPED). The NHANES gathers individual dietary data via a rigorous 24-h dietary recall methodology, integrating it with a questionnaire regarding renal calculi history, thereby establishing a dependable data foundation for examining the diet-lithiasis nexus. The FPED subsequently transforms the raw dietary data into 37 standardized dietary constituents, as delineated by the United States Department of Agriculture, thus transcending the constraints of conventional single-nutrient analyses and empowering researchers to systematically assess the influence of diverse dietary factors on renal calculi risk. Based on the combined NHANES and FPED database, this study aimed to identify key dietary components exhibiting significant associations with KS through the application of a multivariate regression model. This study conducted a comprehensive analysis of dietary determinants in risk of renal calculi, utilizing standardized dietary components, thereby offering novel evidence for elucidating the diet-lithogenesis mechanism. Furthermore, it advocates for a shift in renal calculi prevention guidelines from isolated nutrient restriction to holistic dietary pattern optimization.

## 2 Materials and methods

### 2.1 Study population

This investigation employed data derived from the NHANES and its associated FPED, focusing on individuals aged 20 and above. The NHANES database constitutes a comprehensive and representative multi-stage complex sampling survey, conducting biennial collections of representative samples from the United States populace to enhance comprehension of the nation’s nutritional and health profiles. Data acquisition involved in-person interviews conducted within respondents’ residences, health assessments performed at mobile examination facilities, and analyses of laboratory specimens. The FPED serves to evaluate the conformity of American food and beverage consumption with the recommendations stipulated by the Dietary Guidelines for Americans. It translates food and beverage entries from the What We Eat in America (WWEIA) component of NHANES into 37 distinct food pattern components. This study compiled data spanning from 2007 to 2020, selected for its inclusion of kidney stone history. Owing to the disruptive influence of the pandemic, data from 2017 to 2020 were aggregated. The NHANES protocol received approval from the Institutional Review Board of the National Center for Health Statistics. All participants rendered written informed consent prior to their inclusion within the NHANES database.

### 2.2 Evaluation of KS

Comprehensive data pertaining to kidney stone history are available within the Kidney Conditions-Urology section of the NHANES database. Participants who respond “yes” are categorized as individuals with KS, while those who respond “no” are categorized as non-KS individuals.

### 2.3 Dietary composition assessment

Dietary information was procured through participants’ completion of a validated dietary recall interview. The interviewee’s dietary data, collected over two days, were extracted and subsequently averaged to determine their daily dietary intake. The fruit group encompasses three constituents: citrus, melons, and berries (F_CITMLB); other fruits (F_OTHER); and fruit juice (F_JUICE). The initial two constituents comprise fruits consumed in their whole form or as fruit segments, excluding fruit juices. The fruit juice constituent incorporates both citrus and non-citrus fruit extracts. Total fruit (F_TOTAL) represents the aggregate of all food items within the fruit constituents. The vegetable group comprises five constituents: dark green (V_DRKGR), red and orange (tomatoes (V_REDOR_TOMATO), other red and orange vegetables (V_REDOR_OTHER)), starchy (potatoes (V_STARCHY_POTATO), other starchy vegetables (V_STARCHY_OTHER)), other (V_OTHER), and beans, peas, and lentils (legumes) (V_LEGUMES). Vegetable juices were categorized within their corresponding vegetable groups. Total vegetable (V_TOTAL) represents the aggregate of all food items within the vegetable constituents, excluding beans and peas (legumes). The protein foods group encompasses total meat, poultry, and seafood (PF_MPS_TOTAL); eggs (PF_EGGS); nuts and seeds (PF_NUTSDS); legumes (PF_LEGUMES); and soy products (PF_SOY). Total protein foods (PF_TOTAL) represents the aggregate of all food items within the protein foods constituents, excluding beans and peas. The grains group comprises two constituents: whole grains (G_WHOLE) and refined grains (G_REFINED). Total grains (G_TOTAL) represent the aggregate of all food items within the grain constituents. The dairy group comprises the following constituents: milk (D_MILK), yogurt (D_YOGURT), and cheese (D_CHEESE). Total dairy (D_TOTAL) represents the aggregate of all food items within the dairy constituents. Added sugars are defined as those sugars incorporated into foods as ingredients during preparation, processing, or at the point of consumption. This definition excludes naturally occurring sugars, such as lactose in milk and fructose in whole or cut fruit and 100% fruit juice. Oils encompass all unhydrogenated vegetable oils, excluding palm oil, palm kernel oil, and coconut oil, alongside fats naturally occurring in nuts, seeds, avocados, olives, and seafood. Solid fats encompass those naturally occurring in dairy products, such as milk, cheese, butter, cream, cream cheese, and sour cream; fats naturally present in meat, poultry, and eggs; lard; fully or partially hydrogenated fats and shortenings; cocoa butter; coconut oil; and palm oil. Alcoholic Drinks(A_DRINKS) comprise all varieties of beers, wines, distilled spirits—including brandy, gin, rum, vodka, and whiskey—and cordials and liqueurs. For comprehensive details regarding fruit, vegetable, dairy, protein foods, grains, added sugars and their conversions to cup equivalents (cup equ.), ounce equivalents (oz. eq.), and teaspoon equivalents (tsp. eq.), consult the User Guide for the FPED database (15).

### 2.4 Covariates

Through a comprehensive review of existing literature, we identified and considered the following covariates: Age, sex, marital status (married or living with partner, living alone), race (Mexican American, non-Hispanic Black, non-Hispanic White, other Hispanic, other), education status (under high school, high school or equivalent, above high school), family poverty income ratio (PIR) (<1.3, 1.3 ~ 3.5, > = 3.5), BMI (<25, 25 ~ 30, ≥30), physical activity in recreational time (less than moderate, moderate, vigorous), smoking status (never, former, now), hypertension (yes, no), DM (yes, no).

### 2.5 Statistical analyses

Continuous variables were expressed as means ± standard deviations (SD), and categorical variables were presented as counts (percentages). Disparities between groups were assessed using the chi-square test for categorical variables and the t-test for continuous variables. The associations between the risk of KS and dietary composition were evaluated using binary logistic regression models, with results presented as odds ratios (OR) and 95% confidence intervals (CI). Furthermore, a weighted multivariate logistic regression model was constructed to adjust for age, sex, race, marital status, BMI, physical activity in recreational time, smoking status, hypertension and DM. To ensure the representativeness of our findings, all data were weighted in accordance with NHANES guidelines. The potential for nonlinearity in these statistical relationships was evaluated using restricted cubic spline (RCS) models. Nonlinear examinations encompassed the relationship between total fruit, total vegetable, total protein foods, total dairy, total grains, added sugars, alcoholic beverages, oils, solid fats, and risk of KS. Multicollinearity in the multivariable models was assessed using variance inflation factors (VIFs). All VIFs were less than 5, indicating that multicollinearity was not a significant concern. All analyses were conducted using R Studio version 4.2.2. The ‘nhanesR’, ‘reshape2’, ‘do’, ‘dplyr’, ‘openxlsx’, and ‘survey’ packages were employed for the download, cleaning, and organization of NHANES data. The ‘rms’ and ‘ggplot2’ packages facilitated nonlinear detection and graphical representation. The ‘car’ package is used to perform collinearity checks. We removed the missing data. Statistical significance was defined as *p* < 0.05.

## 3 Results

### 3.1 Population characteristics

**Figure 1:**
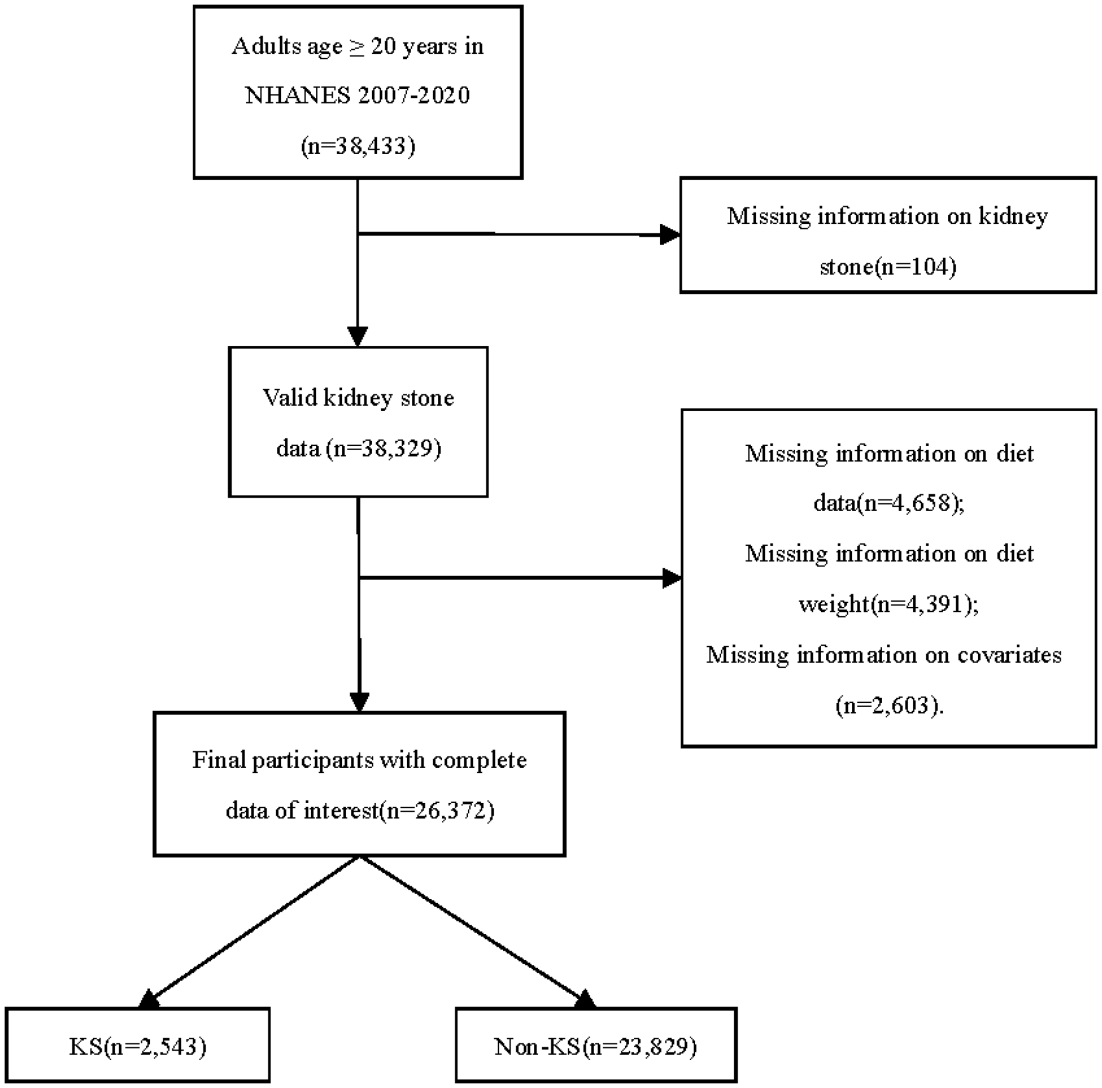
Flowchart detailing participant selection. NHANES, the National Health and Nutrition Examination Survey; KS, kidney stone.

Our investigation centered on NHANES data spanning the years 2007–2008 through 2017–2020. Over this interval, a cumulative total of 38,433 individuals, aged 20 and above, were interviewed and subjected to pertinent examinations within the NHANES database. Of these participants, 38,329 possessed complete records regarding KS history. Among the 26,372 individuals with complete data sets, 2,543 were identified with KS, while 23,829 were categorized as non-KS individuals (Figure 1). The incidence rate of kidney stones is 9.6%. The fundamental characteristics and dietary component intakes of both non-KS and KS groups were delineated within Table 1. Significant associations between the following baseline characteristics and KS were demonstrated by the t-test or chi-square test: age, sex, race, marital status, BMI, physical activity in recreational time, smoking status, hypertension and DM (*p* < 0.05). Statistical analysis revealed significant associations between KS and specific dietary component types (F_TOTAL, F_CITMLB, V_DRKGR, V_STARCHY_POTATO, PF_SOY, D_TOTAL, D_MILK, D_CHEESE and A_DRINKS) (*p* < 0.05).

**Table 1:** General characteristics of the study, categorized according to kidney stone status.

Variables	Total	Non-KS	KS	*p* value
Age (years)	47.38±0.27	46.77±0.28	53.11±0.47	< 0.001
Sex (%)				< 0.001
Female	13815 (52.20)	12670 (52.99)	1145 (44.84)	
Male	12557 (47.80)	11159 (47.01)	1398 (55.16)	
Race (%)				< 0.001
Non-Hispanic White	11495 (67.66)	10058 (66.63)	1437 (77.31)	
Non-Hispanic Black	5841 (10.85)	5500 (11.39)	341 (5.81)	
Mexican American	3578 (8.18)	3283 (8.43)	295 (5.77)	
Other Hispanic	2593 (5.66)	2320 (5.67)	273 (5.54)	
Other	2865 (7.66)	2668 (7.88)	197 (5.57)	
Marital status (%)				< 0.001
living alone	10607 (36.83)	9694 (37.59)	913 (29.73)	
Married/living with partner	15765 (63.17)	14135 (62.41)	1630 (70.27)	
Education status (%)				0.700
Under high school	5628 (14.27)	5067 (14.31)	561 (13.95)	
High school/equivalent	2068 (7.46)	1866 (7.50)	202 (7.14)	
Above high school	18676 (78.27)	16896 (78.20)	1780 (78.91)	
PIR (%)				0.210
<1.3	8149 (21.59)	7386 (21.72)	763 (20.38)	
1.3~3.5	9929 (34.80)	8937 (34.56)	992 (37.07)	
>=3.5	8294 (43.61)	7506 (43.72)	788 (42.55)	
BMI(kg/m2)				< 0.001
<25	7233 (29.20)	6731 (30.03)	502 (21.49)	
25~30	8515 (32.75)	7680 (32.82)	835 (32.12)	
>=30	10624 (38.05)	9418 (37.16)	1206 (46.39)	
F_TOTAL (cup eq.)	0.98±0.01	0.99±0.02	0.92±0.03	0.040
F_CITMLB	0.22±0.01	0.22±0.01	0.19±0.01	0.010
F_OTHER	0.50±0.01	0.50±0.01	0.48±0.02	0.370
F_JUICE	0.26±0.01	0.26±0.01	0.25±0.01	0.370
V_TOTAL (cup eq.)	1.57±0.01	1.57±0.01	1.54±0.04	0.460
V_DRKGR	0.16±0.00	0.16±0.00	0.13±0.01	0.004
V_REDOR_TOMATO	0.30±0.00	0.30±0.00	0.28±0.01	0.110
V_REDOR_OTHER	0.10±0.00	0.10±0.00	0.10±0.01	0.970
V_STARCHY_POTATO	0.36±0.01	0.36±0.01	0.40±0.02	0.030
V_STARCHY_OTHER	0.08±0.00	0.08±0.00	0.08±0.01	0.740
V_OTHER	0.57±0.01	0.57±0.01	0.55±0.03	0.370
V_LEGUMES (cup eq.)	0.12±0.00	0.12±0.00	0.12±0.01	0.870
PF_ TOTAL (oz. eq.)	6.20±0.04	6.19±0.04	6.34±0.13	0.250
PF_MPS_TOTAL	4.80±0.04	4.79±0.04	4.88±0.11	0.390
PF_EGGS	0.56±0.01	0.56±0.01	0.60±0.02	0.130
PF_SOY	0.09±0.00	0.09±0.00	0.07±0.01	0.010
PF_NUTSDS	0.76±0.02	0.75±0.02	0.80±0.05	0.380
PF_LEGUMES (cup eq.)	0.48±0.01	0.48±0.01	0.48±0.04	0.880
D_TOTAL (cup eq.)	1.57±0.02	1.58±0.02	1.44±0.04	< 0.001
D_MILK	0.72±0.01	0.73±0.01	0.65±0.02	< 0.001
D_YOGURT	0.07±0.00	0.07±0.00	0.06±0.01	0.150
D_CHEESE	0.75±0.01	0.75±0.01	0.70±0.02	0.040
G_TOTAL (oz. eq.)	6.44±0.04	6.45±0.04	6.33±0.10	0.280
G_WHOLE	0.91±0.02	0.91±0.02	0.87±0.04	0.340
G_REFINED	5.53±0.04	5.54±0.04	5.46±0.09	0.420
ADD_SUGARS (tsp. eq.)	16.66±0.18	16.59±0.19	17.32±0.41	0.090
A_DRINKS (no. of drinks)	0.67±0.02	0.69±0.02	0.47±0.03	< 0.001
OILS (grams)	25.64±0.20	25.61±0.21	25.95±0.59	0.580
SOLID_FATS (grams)	35.83±0.28	35.76±0.29	36.49±0.64	0.270

KS, kidney stone; F_TOTAL, total fruit; F_CITMLB, citrus, melons, and berries; F_OTHER, other fruits; F_JUICE, fruit juice; V_TOTAL, total vegetables; V_DRKGR, dark green vegetables; V_REDOR_TOMATO, tomatoes; V_REDOR_OTHER, other red and orange vegetables; V_STARCHY_POTATO, potatoes; V_STARCHY_OTHER, other starchy vegetables; V_OTHER, other vegetables; V_LEGUMES, beans, peas, and lentils(legumes); PF_ TOTAL, total protein foods; PF_MPS_TOTAL, total meat, poultry, and seafood; PF_EGGS, eggs; PF_SOY, soy products; PF_NUTSDS, nuts and seeds; PF_LEGUMES, beans, peas, and lentils(legumes); D_TOTAL, total dairy; D_MILK, milk; D_YOGURT, yogurt; D_CHEESE, cheese; G_TOTAL, total grains; G_WHOLE, whole grains; G_REFINED, refined grains; ADD_SUGARS, added sugars; A_DRINKS, alcoholic drinks; cup equ., cup equivalents; oz. eq., ounce equivalents; tsp. eq., teaspoon equivalents; PIR, family poverty income ratio; BMI, body mass index.

### 3.2 Associations of dietary composition with risk of KS

**Figure 2:**
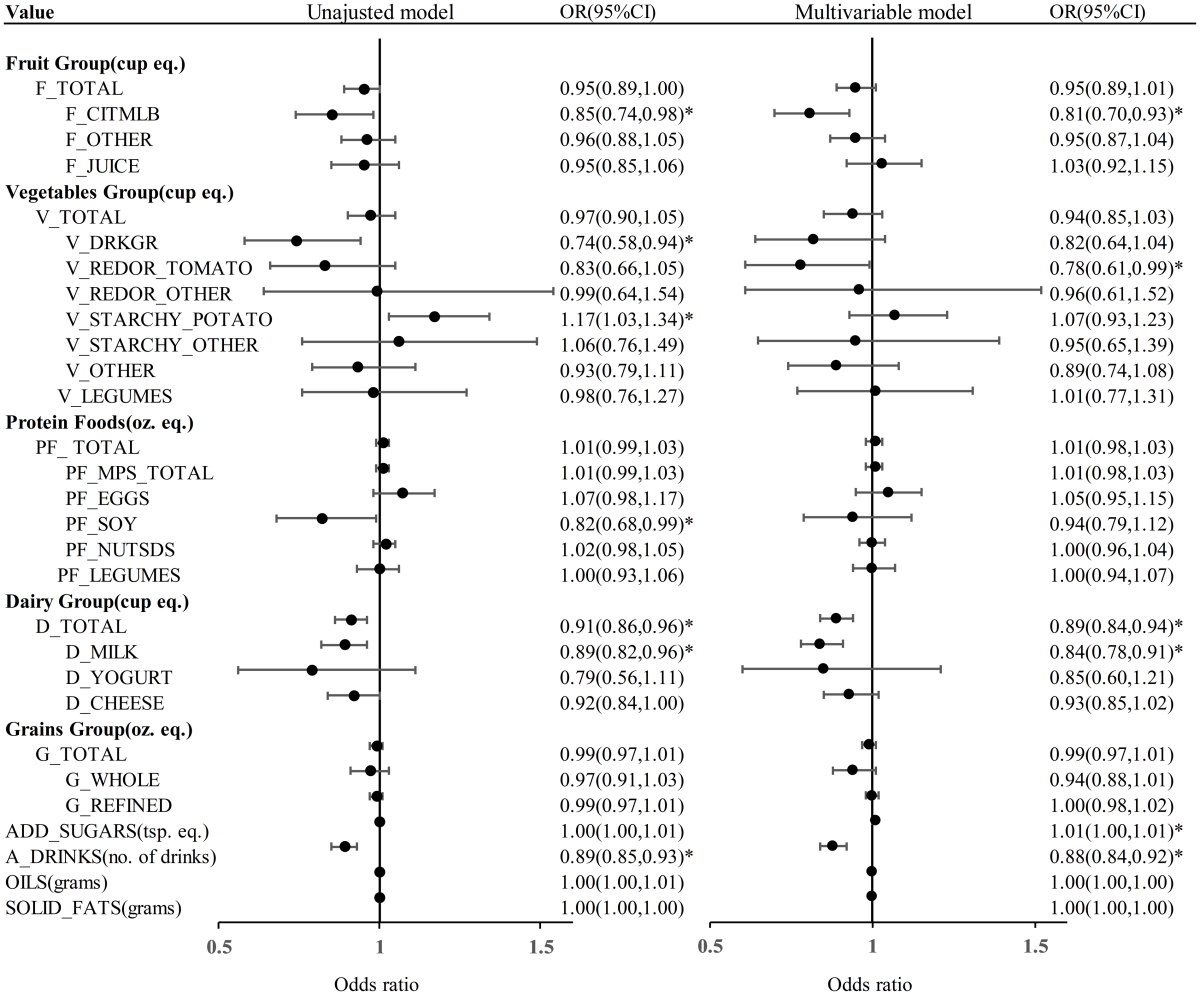
The results of regression analysis models assessing the dietary composition between KS and non-KS. F_TOTAL, total fruit; F_CITMLB, citrus, melons, and berries; F_OTHER, other fruits; F_JUICE, fruit juice; V_TOTAL, total vegetables; V_DRKGR, dark green vegetables; V_REDOR_TOMATO, tomatoes; V_REDOR_OTHER, other red and orange vegetables; V_STARCHY_POTATO, potatoes; V_STARCHY_OTHER, other starchy vegetables; V_OTHER, other vegetables; V_LEGUMES, beans, peas, and lentils(legumes); PF_ TOTAL, total protein foods; PF_MPS_TOTAL, total meat, poultry, and seafood; PF_EGGS, eggs; PF_SOY, soy products; PF_NUTSDS, nuts and seeds; PF_LEGUMES, beans, peas, and lentils(legumes); D_TOTAL, total dairy; D_MILK, milk; D_YOGURT, yogurt; D_CHEESE, cheese; G_TOTAL, total grains; G_WHOLE, whole grains; G_REFINED, refined grains; ADD_SUGARS, added sugars; A_DRINKS, alcoholic drinks; cup equ., cup equivalents; oz. eq., ounce equivalents; tsp. eq., teaspoon equivalents; OR, odds ratio; CI, confidence interval; *p < 0.05. Multivariable model was adjusted for age, sex, race, marital status, BMI, physical activity in recreational time, smoking status, hypertension and DM.

We proceeded to examine the association between diverse dietary components and KS through the application of logistic regression model (Figure 2). In the unadjusted model analysis, daily consumption of citrus, melons, and berries (OR = 0.85, 95% CI = 0.74–0.98), dark green vegetables (OR = 0.74, 95% CI = 0.58–0.94), soy products (OR = 0.82, 95% CI = 0.68–0.99), milk (OR = 0.89, 95% CI = 0.82–0.96), total dairy (OR = 0.91, 95% CI = 0.86–0.96), and alcoholic drinks (OR = 0.89, 95% CI = 0.85–0.93) were associated with a reduced risk of KS development (Table 2). In the unadjusted model analysis, daily consumption of potatoes was associated with an increased likelihood of KS occurrence (OR = 1.17, 95% CI = 1.03, 1.34) (Table 2). The weighted multivariable model analysis revealed that daily consumption of citrus, melons, and berries (OR = 0.81, 95% CI = 0.70–0.93), tomatoes (OR = 0.78, 95% CI = 0.61–0.99), milk (OR = 0.84, 95% CI = 0.78–0.91), total dairy (OR = 0.89, 95% CI = 0.84–0.94), and alcoholic drinks (OR = 0.88, 95% CI = 0.84–0.92) were associated with a lower likelihood of KS development (Table 3). Within the weighted multivariable model analysis, daily consumption of added sugars correlated with an elevated probability of KS occurrence (OR = 1.01, 95% CI = 1.00–1.01) (Table 3). All VIFs were less than 5, indicating that multicollinearity was not a significant concern.

**Table 2:** The results of unadjusted regression analysis models assessing the dietary composition between KS and non-KS.

Variables	OR	95% CI	*p* value
F_TOTAL (cup eq.)	0.95	0.89-1.00	0.050
F_CITMLB	0.85	0.74-0.98	0.020
F_OTHER	0.96	0.88-1.05	0.380
F_JUICE	0.95	0.85-1.06	0.390
V_TOTAL (cup eq.)	0.97	0.90-1.05	0.480
V_DRKGR	0.74	0.58-0.94	0.010
V_REDOR_TOMATO	0.83	0.66-1.05	0.130
V_REDOR_OTHER	0.99	0.64-1.54	0.970
V_STARCHY_POTATO	1.17	1.03-1.34	0.020
V_STARCHY_OTHER	1.06	0.76-1.49	0.730
V_OTHER	0.93	0.79-1.11	0.430
V_LEGUMES	0.98	0.76-1.27	0.880
PF_ TOTAL (oz. eq.)	1.01	0.99-1.03	0.230
PF_MPS_TOTAL	1.01	0.99-1.03	0.380
PF_EGGS	1.07	0.98-1.17	0.120
PF_SOY	0.82	0.68-0.99	0.040
PF_NUTSDS	1.02	0.98-1.05	0.360
PF_LEGUMES	1.00	0.93-1.06	0.880
D_TOTAL (cup eq.)	0.91	0.86-0.96	0.001
D_MILK	0.89	0.82-0.96	0.002
D_YOGURT	0.79	0.56-1.11	0.170
D_CHEESE	0.92	0.84-1.00	0.050
G_TOTAL (oz. eq.)	0.99	0.97-1.01	0.290
G_WHOLE	0.97	0.91-1.03	0.360
G_REFINED	0.99	0.97-1.01	0.430
ADD_SUGARS (tsp. eq.)	1.00	1.00-1.01	0.070
A_DRINKS (no. of drinks)	0.89	0.85-0.93	<0.001
OILS (grams)	1.00	1.00-1.01	0.570
SOLID_FATS (grams)	1.00	1.00-1.00	0.260

F_TOTAL, total fruit; F_CITMLB, citrus, melons, and berries; F_OTHER, other fruits; F_JUICE, fruit juice; V_TOTAL, total vegetables; V_DRKGR, dark green vegetables; V_REDOR_TOMATO, tomatoes; V_REDOR_OTHER, other red and orange vegetables; V_STARCHY_POTATO, potatoes; V_STARCHY_OTHER, other starchy vegetables; V_OTHER, other vegetables; V_LEGUMES, beans, peas, and lentils(legumes); PF_ TOTAL, total protein foods; PF_MPS_TOTAL, total meat, poultry, and seafood; PF_EGGS, eggs; PF_SOY, soy products; PF_NUTSDS, nuts and seeds; PF_LEGUMES, beans, peas, and lentils(legumes); D_TOTAL, total dairy; D_MILK, milk; D_YOGURT, yogurt; D_CHEESE, cheese; G_TOTAL, total grains; G_WHOLE, whole grains; G_REFINED, refined grains; ADD_SUGARS, added sugars; A_DRINKS, alcoholic drinks; cup equ., cup equivalents; oz. eq., ounce equivalents; tsp. eq., teaspoon equivalents; OR, odds ratio; CI, confidence interval.

**Table 3:** The results of multivariable regression analysis model assessing the dietary composition between KS and non-KS.

Variables	OR	95% CI	*p* value
F_TOTAL (cup eq.)	0.95	0.89-1.01	0.080
F_CITMLB	0.81	0.70-0.93	0.004
F_OTHER	0.95	0.87-1.04	0.280
F_JUICE	1.03	0.92-1.15	0.570
V_TOTAL (cup eq.)	0.94	0.85-1.03	0.160
V_DRKGR	0.82	0.64-1.04	0.110
V_REDOR_TOMATO	0.78	0.61-0.99	0.048
V_REDOR_OTHER	0.96	0.61-1.52	0.870
V_STARCHY_POTATO	1.07	0.93-1.23	0.320
V_STARCHY_OTHER	0.95	0.65-1.39	0.800
V_OTHER	0.89	0.74-1.08	0.240
V_LEGUMES	1.01	0.77-1.31	0.960
PF_ TOTAL (oz. eq.)	1.01	0.98-1.03	0.590
PF_MPS_TOTAL	1.01	0.98-1.03	0.650
PF_EGGS	1.05	0.95-1.15	0.370
PF_SOY	0.94	0.79-1.12	0.470
PF_NUTSDS	1.00	0.96-1.04	0.940
PF_LEGUMES	1.00	0.94-1.07	0.960
D_TOTAL (cup eq.)	0.89	0.84-0.94	<0.001
D_MILK	0.84	0.78-0.91	<0.001
D_YOGURT	0.85	0.60-1.21	0.370
D_CHEESE	0.93	0.85-1.02	0.120
G_TOTAL (oz. eq.)	0.99	0.97-1.01	0.500
G_WHOLE	0.94	0.88-1.01	0.110
G_REFINED	1.00	0.98-1.02	0.990
ADD_SUGARS (tsp. eq.)	1.01	1.00-1.01	<0.001
A_DRINKS (no. of drinks)	0.88	0.84-0.92	<0.001
OILS (grams)	1.00	1.00-1.00	0.740
SOLID_FATS (grams)	1.00	1.00-1.00	0.710

F_TOTAL, total fruit; F_CITMLB, citrus, melons, and berries; F_OTHER, other fruits; F_JUICE, fruit juice; V_TOTAL, total vegetables; V_DRKGR, dark green vegetables; V_REDOR_TOMATO, tomatoes; V_REDOR_OTHER, other red and orange vegetables; V_STARCHY_POTATO, potatoes; V_STARCHY_OTHER, other starchy vegetables; V_OTHER, other vegetables; V_LEGUMES, beans, peas, and lentils(legumes); PF_ TOTAL, total protein foods; PF_MPS_TOTAL, total meat, poultry, and seafood; PF_EGGS, eggs; PF_SOY, soy products; PF_NUTSDS, nuts and seeds; PF_LEGUMES, beans, peas, and lentils(legumes); D_TOTAL, total dairy; D_MILK, milk; D_YOGURT, yogurt; D_CHEESE, cheese; G_TOTAL, total grains; G_WHOLE, whole grains; G_REFINED, refined grains; ADD_SUGARS, added sugars; A_DRINKS, alcoholic drinks; cup equ., cup equivalents; oz. eq., ounce equivalents; tsp. eq., teaspoon equivalents; OR, odds ratio; CI, confidence interval. Multivariable model was adjusted for age, sex, race, marital status, BMI, physical activity in recreational time, smoking status, hypertension and DM.

### 3.3 RCS analysis investigating the relationship between risk of KS and dietary composition

**Figure 3:**
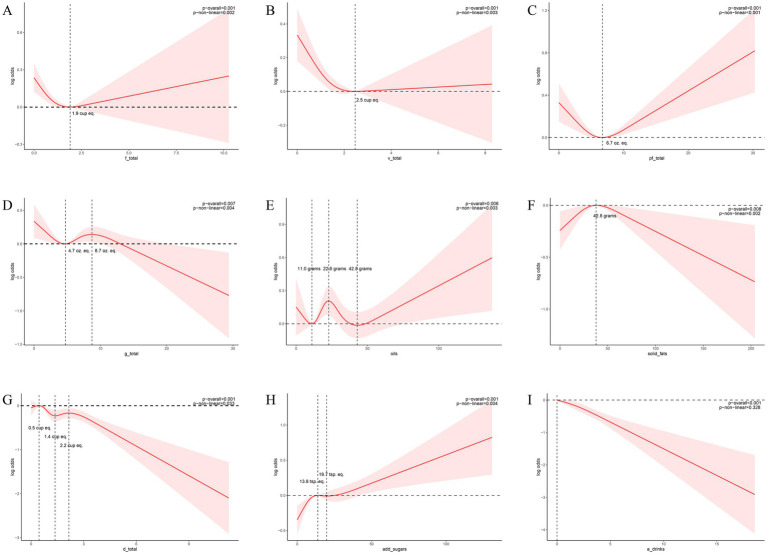
Nonlinear associations between dietary composition and the risk of KS. **(A)** The relationship between total fruit consumption and the risk of KS, as depicted by the RCS model, reveals an inflection point at 1.9 (units: cup eq.). **(B)** The relationship between total vegetable consumption and the risk of KS, as depicted by the RCS model, reveals an inflection point at 2.5 (units: cup eq.). **(C)** The relationship between total protein foods consumption and the risk of KS, as depicted by the RCS model, reveals an inflection point at 6.7 (units: oz. eq.). **(D)** The relationship between total grains consumption and the risk of KS, as depicted by the RCS model, reveals an inflection point at 4.7 and 8.7 (units: oz. eq.). **(E)** The relationship between oils consumption and the risk of KS, as depicted by the RCS model, reveals an inflection point at 11, 22.8 and 42.8 (units: grams). **(F)** The relationship between solid fats consumption and the risk of KS, as depicted by the RCS model, reveals an inflection point at 42.8 (units: grams). **(G)** The relationship between total dairy consumption and the risk of KS, as depicted by the RCS model, reveals an inflection point at 0.5, 1.4 and 2.2 (units: cup eq.). **(H)** The relationship between added sugars consumption and the risk of KS, as depicted by the RCS model, reveals an inflection point at 13.8 and 19.7 (units: tsp. eq.). **(I)** The relationship between alcoholic drinks consumption and the risk of KS, as depicted by the RCS model. Models were adjusted for the following variables: age, sex, race, marital status, BMI, physical activity in recreational time, smoking status, hypertension and DM. The solid red lines correspond to the central estimates, and the red-shaded regions indicate the 95% confidence intervals.

The RCS curve model adeptly illustrated the nonlinear associations between the consumption of total fruits, total vegetables, total protein foods, total dairy, total grains, added sugars, alcoholic beverages, oils, solid fats, and KS (Figure 3). Accounting for the covariates included in the weighted multivariable model, the associations of total fruits, total vegetables, total protein foods, total grains, oils, solid fats, total dairy and added sugars with KS changed at these points (total fruits = 1.9 cup eq., total vegetables = 2.5 cup eq., total protein foods = 6.7 oz. eq., total grains = 4.7 and 8.7 oz. eq., oils = 11.0, 22.8 and 42.8 grams, solid fats = 42.8 grams, total dairy = 0.5, 1.4 and 2.2 cup eq., added sugars = 13.8 and 19.8 tsp. eq.) (p overall and p non-linear <0.05). Nonlinear relationships between the consumption of alcoholic drinks and KS were not observed (p overall < 0.05, p non-linear > 0.05).

## 4 Discussion

Through standardized dietary composition analysis of the NHANES-FPED database, this study observed that citrus, melons, berries, dark green vegetables, tomatoes, milk, alcoholic beverages, and total dairy consumption significantly associated with a diminished renal calculi risk. The consumption of total fruits, vegetables, protein foods, grains, oils, and solid fats exhibited a curvilinear relationship with the risk of renal calculi. The risk of renal calculi diminishes with escalating consumption of total fruits, vegetables, and protein foods, up to 1.9 cup eq., 2.5 cup eq., and 6.7 oz. eq., respectively; conversely, surpassing these thresholds correlates with an augmented risk. Solid fat intake below 42.8 grams precipitates an increased risk, while exceeding this value yields a decreased risk. Total grain consumption within the 4.7–8.7 oz. eq. range elevates lithiasis risk, whereas intakes <4.7 or >8.7 oz. eq. mitigate this risk. Oil consumption <11 grams or 22.8–42.8grams range is associated with a reduction in renal calculi risk as intake increases. Conversely, intakes between 11–22.8 grams, or >42.8 grams, are linked to an increased risk with escalating consumption. Total dairy intake in the range of 0.5–1.4 cup eq. range and >2.2 cup eq. was associated with a reduced risk of KS, while intake below 0.5 cup eq. or between 1.4 and 2.2 cup eq. was associated with an increased risk of KS. Added sugars consumption <13.8 tsp.eq or >19.7 tsp.eq is associated with a reduction in renal calculi risk as intake increases. Conversely, intakes between 13.8–19.7 tsp.eq range, are linked to an increased risk with escalating consumption. Our findings on the protective role of certain fruits and vegetables align with a growing body of evidence suggesting the importance of dietary patterns rich in plant-based foods for urinary health (16). However, unlike previous studies that focused on isolated nutrients or general food groups, our use of the detailed FPED components allows for a more granular analysis. Our analysis revealed a curvilinear, not linear, relationship between the intake of certain food groups—specifically fruit, vegetables, protein, grains, dairy, added sugars, oils, and solid fats—and the risk of KS. This means the risk of developing KS changes after a certain consumption level. Understanding these key inflection points is crucial for better preventing KS formation through dietary choices.

The nonlinear relationships observed for several food groups suggest a complex, dose-dependent effect on KS risk. The inflection points, while derived statistically, may have biological plausibility. The protective association of fruits and vegetables up to a certain threshold may be overcome by the cumulative load of potential promoters (e.g., oxalate) at very high intakes. Similarly, the risk associated with mid-range grain intake could reflect a diet high in refined carbohydrates but lacking the protective fiber of whole grains or the overall caloric moderation of a low-grain diet. The inflection point for solid fats might represent a shift in the balance between pro-inflammatory saturated fats and other dietary components. While these are hypotheses, they underscore that ‘more is not always better’ and highlight the importance of dietary pattern balance over isolated component consumption.

The substantial protective influence of fruit consumption on renal calculi incidence transcends a singular nutrient mechanism. The elevated citric acid concentration in citrus fruits concurrently impedes calcium oxalate lithiasis through dual mechanisms: (1) by complexing with urinary calcium to generate a soluble compound, thereby diminishing calcium oxalate supersaturation; and (2) by directly occluding crystal growth sites, thus retarding the nucleation rate (8, 17–19). The potassium and citric acid found in fruits mitigate the risk of uric acid lithiasis by facilitating uric acid excretion and inducing urinary alkalinization (7, 14). The magnesium content of fruits can competitively inhibit the formation of calcium oxalate crystal lattices (20, 21). Despite the inherent vitamin C content in citrus fruits, which could potentially augment endogenous oxalate synthesis, their observed protective association in this study implies that the oxalate-promoting influence of vitamin C is likely counteracted by the combined action of citric acid and potassium. This finding diverges from the conclusions of a prospective study by Ferraro et al. (22), potentially attributable to the FPED database differentiation between natural fruits and vitamin C supplements, wherein the latter is more predisposed to eliciting supraphysiological oxalate production. Fruit juice, categorized as a distinct ingredient from whole fruit, may attenuate the protective association due to the removal of fiber and the concentration of sugars (10, 23, 24). Oxalate in vegetables is the main component of stone formation. The oxalic acid present in dark green vegetables predominantly exists as insoluble calcium salts, exhibiting an intestinal absorption rate of merely 5–15%, significantly lower than that of pure oxalate supplements (25). Concurrently, the calcium, magnesium, and dietary fiber inherent in vegetables can further impede the absorption of free oxalic acid, thus forming a natural anti-lithogenic complex (10, 21, 26). Tomatoes, abundant in antioxidants (lycopene), may mitigate renal tubular damage via antioxidant mechanisms, and may downregulate oxalate-induced inflammatory signaling pathways within renal epithelial cells, thereby impeding the upstream initiators of lithogenesis (27–29). The absence of differentiation between raw and cooked vegetables (e.g., blanching, which reduces oxalic acid by 40–80%) in our study may have resulted in an underestimation of risk (30). Subsequent analyses, incorporating culinary data, are necessary. Furthermore, the protective associations of these vegetables may be partly mediated by bioactive compounds like flavonoids, which possess potent anti-inflammatory and antioxidant properties that could mitigate renal cellular damage and crystal formation (31). The non-linear relationship observed for fruit indicates a complex, dose-dependent effect on kidney stone (KS) risk. This suggests that while fruit consumption within a specific range may be beneficial and protective, these positive effects could be negated by the cumulative load of potential promoters—such as vitamin C, fructose, and oxalate—at very high levels of intake.

The observed protective associations of milk and total dairy consumption reinforce the stone-protective role of dietary calcium, though the underlying mechanisms extend beyond the simplified model of calcium oxalate binding. The consumption of dairy-derived calcium during meals facilitates its preferential binding to intestinal oxalic acid, thereby diminishing its absorption (32, 33). The ingestion of calcium supplements on an empty stomach may augment urinary calcium excretion (32). Whey protein, abundant in cysteine, elevates glutathione levels, thus attenuating oxidative stress-induced renal tubular cell damage and diminishing calcium oxalate crystal aggregation (34).

Excessive consumption of added sugars can induce insulin resistance, resulting in diminished renal calcium reabsorption and elevated urinary calcium excretion, consequently augmenting the risk of calcium oxalate lithogenesis (35). This study differentiated between added sugars and naturally occurring sugars, thereby mitigating the classification confounding bias inherent in prior investigations.

Our analysis found an inverse association between alcoholic drink consumption and kidney stone prevalence. However, this finding must be interpreted with extreme caution. The effect of alcohol is likely J-shaped and highly dependent on dose, beverage type, and hydration status. While low-to-moderate intake may induce diuresis and potentially dilute urine (36), heavy consumption is a known risk factor for dehydration and impaired renal function, which can promote stone formation (37). The American Urological Association guidelines advise restriction of alcohol for stone prevention (38). Our results, which likely reflect patterns of light-to-moderate consumption within this cohort, should not be construed as an endorsement of alcohol intake for stone prevention. The potential risks of heavy drinking far outweigh any speculative benefits suggested by this associative finding.

Excessive animal protein consumption can elevate urinary excretion of uric acid, calcium, and oxalate, while concurrently lowering urine pH via acidic metabolites, thereby promoting the formation of uric acid and cystine calculi (39–41). A balanced protein intake may preserve urinary electrolyte homeostasis while supplying sufficient cystine inhibitors to impede renal calculi crystal formation. The elevated glycemic index of refined grains may exacerbate insulin resistance, thereby promoting urinary calcium excretion (40). Concurrently, the phytic acid present in certain grains, when consumed in excessive quantities, can impede mineral absorption (e.g., magnesium), indirectly diminishing the body’s natural resistance to lithiasis (42). Moderate consumption of whole grains can furnish dietary fiber and magnesium, which synergistically impede calcium oxalate crystallization and alkalinize urine. Essential fatty acid deficiency may exacerbate inflammatory responses, promote renal tubular epithelial damage, and facilitate the formation of the stone matrix (43). Excessive fat consumption may elevate urinary calcium excretion via oxidative stress and insulin resistance, while high caloric intake leading to obesity indirectly augments the risk of lithiasis (44, 45). A moderate intake of unsaturated fatty acids can exert anti-inflammatory effects, maintain cellular membrane stability, and enhance calcium metabolism equilibrium (46).

This study does possess certain limitations. This study’s cross-sectional design precludes the establishment of causal relationships. Furthermore, the absence of data regarding critical metabolites, such as urinary calcium, oxalate, and citrate, impedes direct validation of the dietary intake to urinary calculi pathway. Future studies incorporating 24-h urine collections would be valuable to elucidate the mediating role of these metabolites and strengthen the causal inference between dietary patterns and kidney stone formation. Additionally, the reliance on self-reported history of kidney stones and two 24-h dietary records may introduce recall bias into our study’s findings. Consequently, we intend to explore more robust assessment methodologies in subsequent investigations, such as collecting kidney stone diagnoses from medical records and gathering 24-h dietary records on multiple, non-consecutive days to enhance reliability. The potential for selection bias due to missing participant data may compromise the interpretation of the findings. Although our study accounted for several key confounding factors, data limitations precluded the inclusion of other potential confounders, such as a familial history of kidney stones. The omission of these variables may have impacted the observed associations; thus, it is imperative for future investigations to endeavor to incorporate such factors. This study conducted exploratory analyses of various dietary components, involving numerous comparisons, but without correction for multiple testing, which increases the risk of false positive results. Therefore, our results should be interpreted as generating hypotheses for future research.

## 5 Conclusion

In conclusion, this cross-sectional study elucidates intricate curvilinear associations between dietary components and the risk of KS. We observed that the intake of citrus, melons, berries, tomatoes and milk was associated with a lower risk of KS. While our results advocate for a shift in preventive guidelines toward holistic dietary patterns, they must be interpreted with caution due to the limitations of the cross-sectional design. Future longitudinal studies are necessary to establish causality.

## Data Availability

The datasets presented in this study can be found in online repositories. The names of the repository/repositories and accession number(s) can be found below: the authors uploaded the data and R code to https://www.figshare.com/ (https://doi.org/10.6084/m9.figshare.28690424).
